# Early extracorporeal membrane oxygenation as bridge for central airway obstruction patients caused by neck and chest tumors to emergency surgery

**DOI:** 10.1038/s41598-023-30665-1

**Published:** 2023-03-06

**Authors:** LianJing Liang, ShiTong Su, YaRong He, YaLan Peng, ShuYun Xu, Yang Liu, YaXiong Zhou, HaiFang Yu

**Affiliations:** 1 Emergency Medicine Department, Emergency Medical Laboratory, West China Hospital, Sichuan University, Chengdu, 610041 China; 2 Department of Head and Neck Oncology, Cancer Center and State Key Laboratory of Biotherapy, West China Hospital, Sichuan University, Chengdu, 610041 China; 3 Medical General Department of Medical Affairs Division, West China Hospital, Sichuan University, Chengdu, 610041 China; 4 Day Surgery Center, West China Hospital, Sichuan University, Chengdu, 610041 China

**Keywords:** Oncology, Surgical oncology, Signs and symptoms, Hypoxia, Respiratory signs and symptoms

## Abstract

Central airway obstruction caused by neck and chest tumors is a very dangerous oncological emergency with high mortality. Unfortunately, there is few literature to discuss an effective way for this life-threating condition. Providing effective airway managements, adequate ventilation and emergency surgical interventions are very important. However, traditional airway managements and respiratory support has only limited effect. In our center, using extracorporeal membrane oxygenation (ECMO) as a novel approach to manage patient with central airway obstruction caused by neck and chest tumors has been adopted. We aimed to show the feasibility: using early ECMO to manage difficult airway, provide oxygenation and support surgical procedure for patients with critical airway stenosis caused by neck and chest tumors. We designed a single-center, small sample size retrospective study based on real-world. We identified 3 patients with central airway obstruction caused by neck and chest tumors. ECMO was used to ensure adequate ventilation to emergency surgery. Control group cannot be established. Because traditional manner very likely led to death of such patients. Details of clinical characteristics, ECMO, surgery and survival outcomes were recorded. Acute dyspnea and cyanosis were the most frequent symptoms. All patients (3/3) showed descending arterial partial pressure of oxygen (PaO_2_). Computed tomography (CT) revealed severe central airway obstruction caused by neck and chest tumors in all cases (3/3). All patients (3/3) had definite difficult airway. All cases (3/3) received ECMO support and emergency surgical procedure. Venovenous ECMO was the common mode for all cases. 3 patients weaned off ECMO successfully without any ECMO-related complications. Mean duration of ECMO was 3 h (range: 1.5–4.5 h). Under ECMO support, difficult airway management and emergency surgical procedure were finished successfully for all cases (3/3). The mean ICU stay was 3.3 days (range: 1–7 days), and the mean general ward stay was 3.3 days (range: 2–4 days). Pathology demonstrated the tumor dignity for 3 patients including 2 malignant cases and 1 benign case. All patients (3/3) were discharged from hospital successfully. We showed that early ECMO initiation was a safe and feasible approach to manage difficult airway for patients with severe central airway obstruction caused by neck and chest tumors. Meanwhile, early ECMO initiation could ensure security for airway surgical procedure.

## Introduction

Clinically, central airway obstruction caused by neck and chest tumors is very dangerous oncological emergency^1,2^, and difficult to manage for emergency departments (ED)^3^. Complex anatomical location between trachea and tumors could result in difficult airway^4^. Acute dyspnea is the most apparent symptom to such patients. Hypoxemia and even asphyxia are major cause of death. Providing effective airway managements and emergency surgical interventions is the key to save these patients^5,6^.

Traditional airway managements, including nasal oxygen inhalation, respiratory mask, implanted hard and soft endoscopy, laryngeal mask, tracheal intubation, and even tracheotomy, are only suitable for patients with mild obstructed airway^7^. Traditional approaches usually have only limited effect and even life-threating for patients with critical obstructed airway^3,8^. Severe central airway obstruction caused by neck and chest tumors show high mortality^7–10^. Direct compression of adjacent tumor is the most common cause^11,12^. Providing adequate ventilation (CO_2_ removal and oxygenation) and emergency surgical interventions can play a significant role to manage such patients^13,14^.

Extracorporeal membrane oxygenation (ECMO) has been used to manage cardiac and respiratory failure for more than 40 years. It has also emerged as a useful cares of short-term support for hypoxic patients with nontraditional indications, such as upper airway surgery^6,15,16^, lung transplantation^17^, pulmonary embolism^18^ and airway foreign body^19^. ECMO can help to ensure adequate ventilation when performing surgical therapies^20^. American Society of Anesthesiologists (ASA) presented an updated version of the *Practice Guidelines for Management of Difficult Airway* in 2022. Compared with 2013 version^21^, ECMO as a novel significant device, has been recommended for managing difficult airway^22^. However, there is few report of early ECMO initiation to manage difficult airway and provide adequate ventilation for such patients with central airway obstruction caused by neck and chest tumors. We study and present the availability of early ECMO initiation as a bridge for these patients to emergency surgery operations.

## Methods

All methods were performed in accordance with the relevant guidelines and regulations, and approved by the Ethics Committee on Biomedical, West China Hospital of Sichuan University (Number: 2021-233). It was a single-center, retrospective small sample study based on real-world. Informed consents were obtained from all patients when they were admitted to our emergency center. The center of West China Hospital, Sichuan University had professional ECMO team and respiratory ICU.

Traditional respiratory supports had extremely limited effect for severe central airway obstruction patients caused by neck and chest tumors.

To explore feasibility of early ECMO initiation as an effective first-aid manner for these patients, we reviewed clinical records of patients between January 2021 and December 2021. Severe central airway obstruction caused by neck and chest tumors, unfeasible traditional respiratory supports and early ECMO intervention were eligible for inclusion. Patients were selected by our respiratory MDT, based on criteria of *Practice Guidelines for Management of Difficult Airway (2022 version)*, presented by American Society of Anesthesiologists (ASA)^21,22^. 3 patients were completely eligible for inclusion standard. There was no control group. Because it was life-threating for severe central airway obstruction patients caused by neck and chest tumors to use traditional manner. Establishing adequate ventilation was safest for patients. Therefore, we were unable to set control group with traditional manner to compare with ECMO group. We obtained the demographic characteristics, clinical features, blood tests, radiological managements, surgical procedures, pathological examinations, ECMO details and survival outcomes to make a true presentation. Presenting how to build emergency ventilation for severe central airway obstruction patients caused by neck and chest tumors was our primary objective. Central airway obstruction caused by neck and chest tumors is very dangerous oncological emergency with increasing incidence. Discussing an effective first-aid plan to save their life was our secondary objective.

In this part, we showed detailed clinical experience to make that every center could repeat this procedure in the same manner. The primary step was central airway obstruction caused by neck and chest tumors verified by CT performed before treatment. The CT outcomes were interpreted by experienced radiologist or emergency physician. Evaluation and management of difficult airway were performed by anesthesiologist at patient bedside. If traditional managements was useless and even life-threating, ECMO as a significant device could be recommend to provide adequate ventilation. Under ECMO support, surgical procedures were carried out. Conflict between anticoagulation and surgical bleeding should be pay attention. Pharmacokinetics of heparin was important. Kidney played a key role in heparin clearance. Renal function test was significant before using heparin. In our center, firstly, heparin-free was attempted when ECMO was running to provide adequate ventilation. Secondly, coagulation function was tested until reaching surgical standard. Thirdly, surgical procedures were performed during ECMO running without heparin. Lastly, anticoagulation was restarted after operations with acceptable surgical bleeding. All details and variables were recorded.

## Results

**Figure 1:**
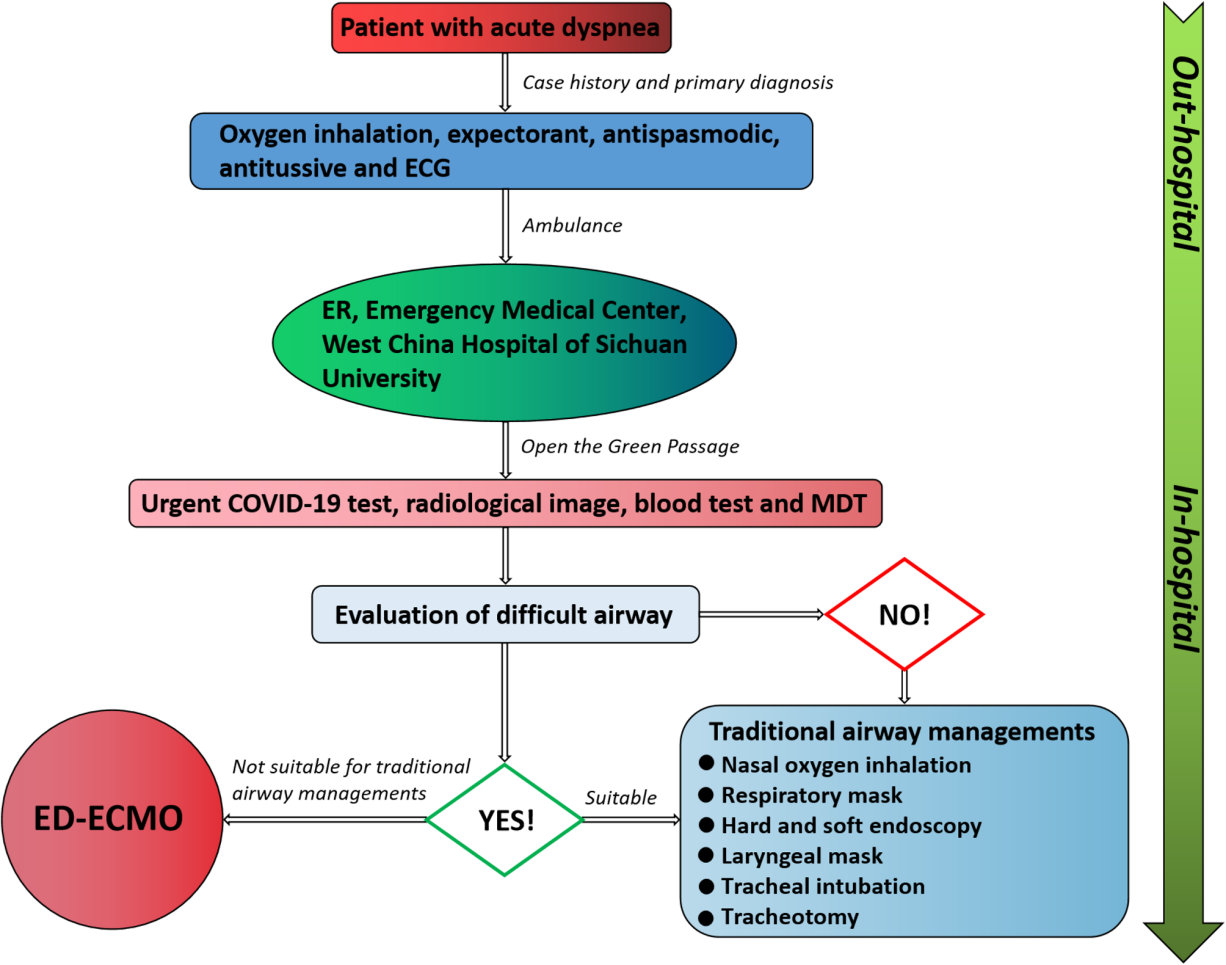
Green passage for patient with possible difficult airway. ED, emergency departments; ER, emergency room; ECG, electrocardiogram; ECMO, extracorporeal membrane oxygenation; MDT, multidisciplinary team.

3 patients with severe central airway obstruction caused by neck and chest tumors were admitted to our center, who received early ECMO support with the Green Passage (Fig. 1).

Of 3 patients (Table 1), 1 was male, 2 were females. The mean age was 39 (range, 18–52) years. Acute dyspnea was common symptom for all cases. 2 patients presented cyanosis of lips and three concave sign.

**Table 1:** Clinical characteristics and treatments of patients.

Case/age/sex/ER and admission	Clinical picture	CT diagnosis	Evaluation and management of difficult airway
1/52/M 06/01/2021	Dyspnea, Cyanosis, TCS and Chest pain	Central airway obstruction, critical airway stenosis, multiple enlarged medicinal lymph nodes, thickening of thyroid isthmus	Yes/oxygen inhalation, expectorant, antispasmodic, antitussive, early ECMO initiation and emergency surgery
2/18/F 14/11/2021	Dyspnea and Chest pain	Mediastinal mass with maximum diameter in 88 mm, central airway obstruction, critical airway stenosis, right brachiocephalic vein compression	Yes/oxygen inhalation, early ECMO initiation and emergency surgery
3/46/F 22/12/2021	Dyspnea, cyanosis, TCS and fever	Thickening of carina, central airway obstruction, critical airway stenosis	Yes/oxygen inhalation, expectorant, antispasmodic, antitussive, anti- infection, early ECMO initiation and emergency surgery

ER, emergency room; TCS, three concave sign; CT, computed tomography; ECMO, extracorporeal membrane oxygenation.

**Figure 2:**
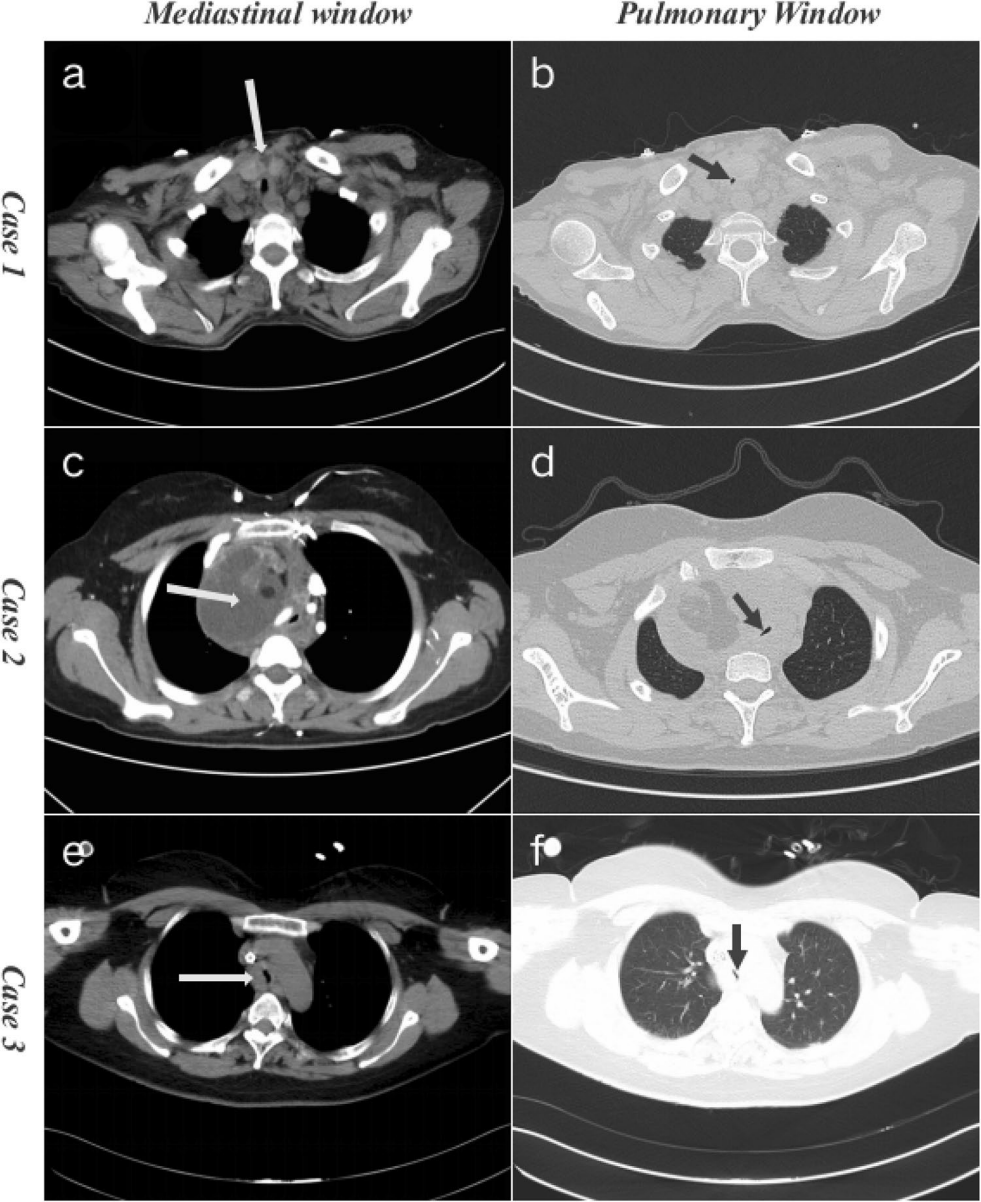
CT scans of severe central airway obstruction patients caused by neck and chest tumors before ECMO (Axial view). White arrow shows tumors. Black arrow shows severe central airway obstruction. Thickening of thyroid isthmus, multiple enlarged neck lymph nodes and critical airway stenosis of Case 1 (**a**, **b**). Mediastinal huge mass and critical airway stenosis of Case 2 (**c**, **d**). Thickening of carina and critical airway stenosis of Case 3 (**e–f**).

2 patients showed chest pain. 1 patient had fever. CT scan was performed in 3 patients with imaging abnormal findings. In total, critical central airway stenosis was obvious diagnosis for 3 patients. In detail, case1 was believed to have been caused by neck tumor, case 2 by mediastinal tumor of chest, and case 3 by airway tumor of chest (Fig. 2).

3 patients (100%) were diagnosed with critical central airway stenosis caused by neck and chest tumors. Acute dyspnea prompted 3 patients to receive adequate ventilation with ECMO. Because traditional respiratory support was life-threating for them (Table 1).

Of note, 3 patients expressed severe respiratory symptoms and did thus undergo arterial blood gas analysis. Descending arterial partial pressure of oxygen (PaO_2_) value revealed respiratory failure in all cases (Table 2).

**Table 2:** Arterial blood gas analysis.

Arterial blood gas analysis	Local reference	Case 1	Case 2	Case 3
pH value	7.35–7.45	7.432	7.452	7.419
PaO_2_	80–100 mmHg	72.6	75.3	68.4
PaCO_2_	35–45 mmHg	45.2	33.0	31.8
HCO_3_-	22–27 mmol/L	28.9	23.7	20.1
BE	− 2 to + 3 mmol/L	4.0	− 0.8	− 3.4
AG	12–20 mmol/L	7.4	11.7	11.4

PaO_2_, arterial partial pressure of oxygen; PaCO_2_, arterial partial pressure of carbon dioxide; BE, base excess; AG, anion gap.

All patients (3/3) were subjected to peripheral venovenous extracorporeal membrane oxygenation (VV ECMO). Femoral and internal jugular vein (FJV) was common cannulation site in 3 patients. Heparin was used as anticoagulant during early ECMO run for all patients. Mean duration of ECMO was 3 h (range: 1.5–4.5 h). All patients were successfully weaned from ECMO without relevant complications (Table 3).

**Table 3:** Details of early ECMO initiation.

Details of ECMO	Case 1	Case 2	Case 3
Indication for ECMO	Critical airway stenosis caused by thickening of thyroid isthmus and multiple enlarged lymph nodes	Central airway obstruction caused by compression and extension from adjacent mass	Thickening of trachea and carina, critical airway stenosis
Mode of ECMO	VV	VV	VV
Cannulation site	FJV	FJV	FJV
Anticoagulation	Heparin	Heparin	Heparin
Start Time of ECMO	Definite difficult airway in ER	Definite difficult airway in ER	Definite difficult airway in ER
Early ECMO Run time (hours)	1.5	4.5	3
Weaning off ECMO	Successful	Successful	Successful

ECMO, extracorporeal membrane oxygenation; VV, venovenous; FJV, femoral and internal jugular vein; ER, emergency room.

All patients received surgical operations (Table 4). In detail, 3 patients received mass removal and postoperative assisted ventilation (PAV). Of these, 2 patients received tracheal reconstruction. The mean PAV time was 2.7 days (range: 1–5 days), and no case required prolonged mechanical ventilation due to complications. The mean ICU stay was 3.3 days (range: 1–7 days), and the mean general ward stay was 3.3 days (range: 2–4 days). There was no in-hospital death after surgery.

**Table 4:** Surgical details of patients.

Details of ES	Case 1	Case 2	Case 3
Surgical procedures	Thyroid isthmus and airway mass removal, Neck lymph node dissection, tracheal reconstruction	Mediastinal mass removal by thoracotomy	Airway mass removal and tracheal reconstruction by thoracotomy
Intraoperative findings	Central airway compressed and invaded by infiltrating thyroid neoplasm, multiple metastasis of neck lymph nodes	Severe critical airway stenosis caused by huge mediastinal mass with clear boundary and cystic-solid profile	Central airway obstruction caused by infiltrating airway mass and thickening carina
Intraoperative diagnosis	Central airway invasion and multiple lymph node metastasis from malignant tumor of thyroid isthmus	Huge benign tumor of mediastinum	Malignant tumor of Central airway
ICU (days)	1	2	7
PVA (days)	1	2	5
General ward (days)	2	4	4
Surgical pathology	Thyroid follicular carcinoma	Mediastinal mature cystic teratoma	Adenoid cystic carcinoma of airway
Tumor dignity	Malignant	Benign	Malignant
Survival	Alive	Alive	Alive

ES, emergency surgery; ICU, intensive care unit; PAV, postoperative assisted ventilation.

**Figure 3:**
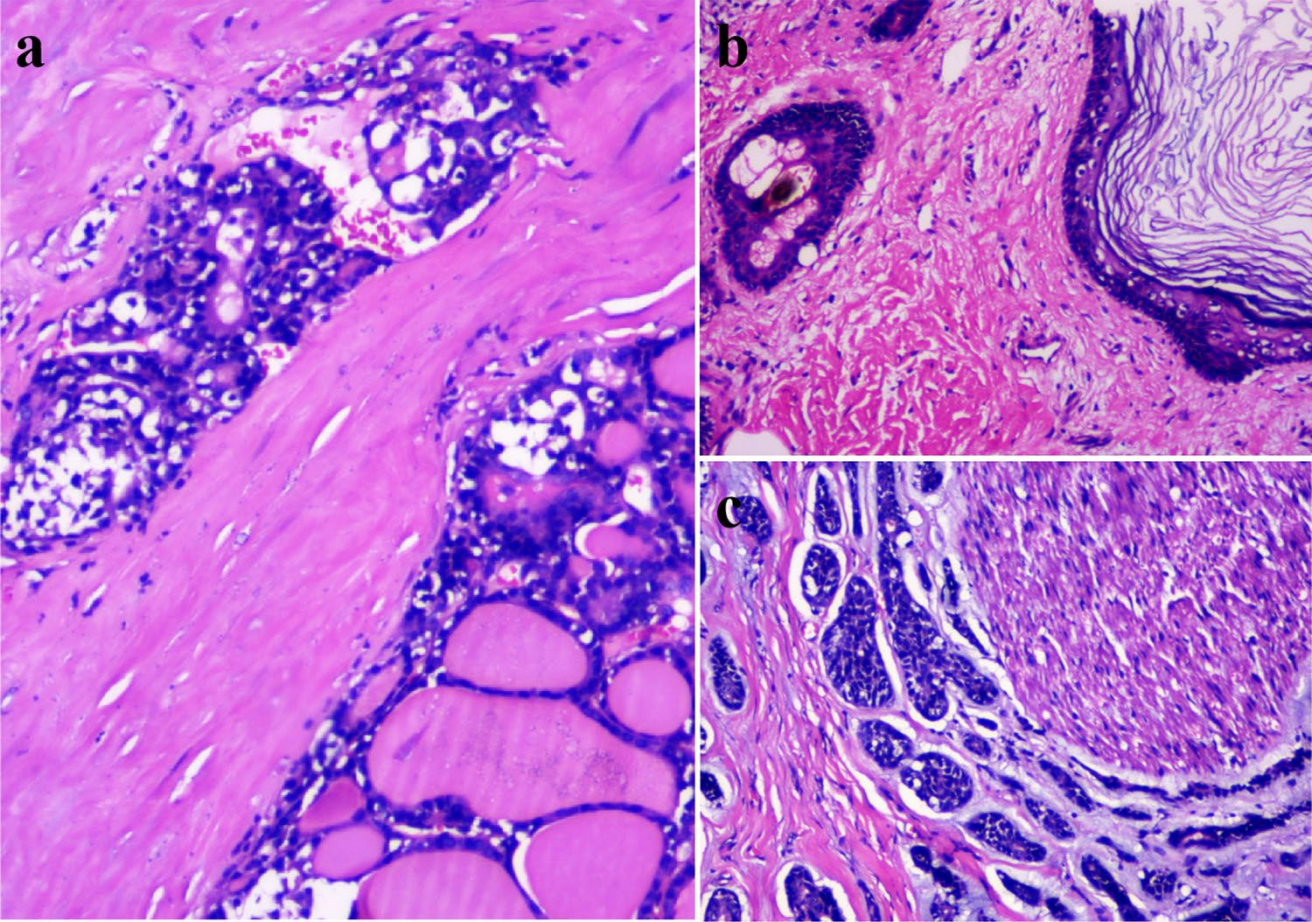
Pathological examination of the tumor dignity (H & E staining, × 100). Thyroid follicular carcinoma of Case 1 (**a**). Mature cystic teratoma of Case 2 (**b**). Adenoid cystic carcinoma of Case 3 (**c**).

Pathology (Fig. 3) demonstrated the tumor dignity including 2 malignant cases (thyroid follicular carcinoma of Case1 and adenoid cystic carcinoma of Case 3) and 1 benign case (mediastinal mature cystic teratoma of Case 2). All patients (3/3) were successfully discharged from hospital.

## Discussion

Central airway obstruction is dangerous emergency. Airway oppressed by adjacent tumors show high mortality with limited treatments^1,2^. Asphyxia and respiratory failure are main causes of death in such patients^3,7–10^. For these patients with a rapid decline in respiratory status, they must be stabilized before effective treatments^17^. Effective airway managements are very significant for them^23–25^. Respiratory support to optimize oxygenation is an important issue for critical airway obstruction^26,27^. When the airway becomes narrowed, the life of the patients is threatened because of impending suffocation. Traditional respiratory supports can be performed quickly^11,12^. However, in emergency, the traditional respiratory support treatment may be life-threating for patients with severe central airways obstruction caused by neck and chest tumors. Because traditional procedures possibly lead to complete airway obstruction and even death of patients^14^. Effective airway managements and active surgical intervention are the key to save life of patients^11,12,21,22^. Although, the process of management and treatment is different to each medical center. For patients with severe central airway obstruction, the thorough knowledge of the pathology, physiology, diagnostic, and therapy options are required with a multidisciplinary team (MDT) approach.

In recent years, the stent placement as an interventional way to palliate the airway obstruction caused by tumor is becoming widely accepted^7,8,10–12^. Because stent placement is safe, minimally invasive, well tolerated, and is increasingly used in various benign and malignant conditions characterized by central airway stenosis^13,14,26,28^. However, in our cases, using stent is very dangerous. There are two reasons. On the one hand, without adequate ventilation, stent placement can lead to complete obstruction for severe airway stenosis patients. On the other hand, stent touch tumor that may lead to major bleeding because of high intensive vascularity of tumor. For our patients, the mean age is only 39 years. Although, patients can gain short palliation from stent placement, surgical operation is the most effective way to treat tumors. In addition, stent placement may have some unexpected complications, such as stent migration^28–31^, fracture^28,31^, falling off, deformation^28^, tracheal stent buckling and in-stent stenosis^32^.

Providing adequate ventilation to support surgical operations is feasible for the 3 patients. However, traditional manners cannot ensure adequate ventilation. Firstly, tracheal intubation is unable to pass the narrow location of severe airway stenosis. Secondly, tracheostomy have high risk of major bleeding because of adjacent tumor with high intensive vascularity. Descending PaO_2_ value and symptom of hypoxia revealed respiratory failure in our cases. When traditional manner is unfeasible, to ensure adequate ventilation for unstable patients, we used ECMO to manage difficult airway and support surgical procedure.

Previous clinical studies showed that ECMO could be used to ensure adequate ventilation for stent placement in patients with critical airway obstruction^13,14,28^. However, for severe central airway obstruction patients caused by neck and chest tumors, literature of early ECMO initiation as a bridge to support surgery is rare^5^. ECMO as a significant device to manage severe central airway obstruction patients caused by neck and chest tumors still need further clinical practice to demonstrate it.

In our study, all patients received early ECMO support with mode of venovenous extracorporeal membrane oxygenation (VV ECMO).

In brief, VV ECMO, cannulation site from femoral vein to internal jugular vein, was preferred for respiratory failure in thoracic surgeries. Previous studies have reported that VV ECMO can help to ensure adequate ventilation (CO_2_ removal and oxygenation) when performing stent placement in patients with central airway obstruction^13,14,28^. VV ECMO can provide time to plan and implement adequate treatment, thereby minimizing procedure-related complications^28^. Furthermore, operating physician feel more comfortable using VV ECMO when performing a high-risk procedure^14^. There is also clinical study that presented using venoarterial ECMO (VA ECMO) in patients with airway problems^20^. VA ECMO, cannulation site from femoral vein to femoral artery, was used usually when patients need circulatory support based on clinical and echocardiogram assessment in addition to respiratory support. The VA approach is recommended if the patient show cardiac arrest or shock, whereas the VV technique may be sufficient when only ventilation and oxygenation are required. Compared with VV ECMO, the VA ECMO reduces pulmonary blood flow and is associated with a higher incidence of neurologic events (eg, cerebral infarction, microembolism, or hemorrhage), cognitive impairment, and major local complications such as arterial dissection, pseudoaneurysm formation, or limb ischemia^5,20^. For our patients, the VV ECMO may be more suitable than VA ECMO to ensure adequate ventilation for airway management and emergency surgery. ECMO-related complication rates of 24–55% have been reported, including potentially life-threatening complications such as bleeding, hemolysis, air leakage and thrombosis^28,33,34^. We used Heparin to guard against thrombosis. All cases received coagulation test. We should pay attention to conflict between anticoagulation and surgical bleeding. Finishing operations within time window from Heparin-free staring to membrane blood coagulation appearing is very important.

In our cases, the mean duration of early ECMO was 3 h (range: 1.5–4.5 h), our mean duration was shorter than 15 ± 18 h (range: 1–51 h)^5^, 20.9 h (range: 2.2–113.4 h)^14^ and 42 h (range: 5–121 h)^28^ of published literatures. And there was no any ECMO-related complication in our all cases from early ECMO initiation to weaning off ECMO successfully. This results may associate with short running time of ECMO and adequate prevention of ECMO-related complications. Of note, ECMO-related complications are closely related to the technical proficiency and experience of the operating physician.

Surgery is most effective treatment for tumor disorder. All cases were subjected to surgical procedure. We showed a shorter mean in-hospital time than published papers^5,14,28^. Hypoxia may increase the potential risk for cardiovascular, pulmonary, central nervous injuries and surgical intolerance. In our study, 2 patients showed chest pain that may revealed the subtler myocardial damage. Early ECMO initiation may protect cardiopulmonary function from hypoxia to increase surgical tolerance. 3 patients were successfully discharged from hospital (Supplementary file).

Interpretation of our results is limited by the single-center, small sample study. There are no comparisons between groups. So, we cannot know for certain whether our surgical procedure could have been accomplished without ECMO support. Setting control group is limited by two reasons. Firstly, traditional manner is unfeasible and even life-threating for patients. Secondly, to compare a lower-risk airway cases would expose the study to selection bias.

The sample size is very small (n = 3), any meaningful comparison would be limited. Although ASA recommended ECMO as a significant device to manage difficult airway in 2022^22^. The paper of using early ECMO to manage severe central airway obstruction patients caused by neck and chest tumor is rare. With increasing number of these patients, when traditional way may be unfeasible and dangerous, finding an effective first-aid is very important. Our study purpose was to discuss the feasibility of using ECMO as a bridge for central airway obstruction patients caused by neck and chest tumors to emergency surgery operations.

However, grass-roots hospitals and some third-level hospitals lack the equipment and operating experience of ECMO, it could be extremely dangerous to transport such patients. So, ECMO-related teaching should be improved. And our center has assigned ECMO groups to help these hospitals. In addition, we have operated prehospital ECMO procedure to transport such patients to our center with professional ECMO team and equipment.

## Conclusions

Early ECMO initiation as a bridge can be used to manage difficult airway, provide oxygenation and support surgical procedure for patients with critical central airway stenosis caused by neck and chest tumors. Early ECMO initiation can provide safety and security for airway surgical procedure. Our report further demonstrated the feasibility of ECMO as a significant device to save life of such patients in emergency (Supplementary file).

## Supplementary Information

Supplementary Information 1.

Supplementary Information 2.

Supplementary Information 3.

Supplementary Information 4.

Supplementary Information 5.

Supplementary Information 6.

## Data Availability

All data generated or analyzed during this study are included in this published article.

## Abbreviations

ED: Emergency departments
ER: Emergency Room
ECG: Electrocardiogram
ECMO: Extracorporeal membrane oxygenation
ASA: American Society of Anesthesiologists
CT: Computed tomography
PaO_2_: Partial pressure of oxygen
FJV: Femoral and internal jugular vein
ICU: Intensive care unit
PAV: Postoperative assisted ventilation
MDT: Multidisciplinary team
