# Supplementary figures and images for: Early extracorporeal membrane oxygenation as bridge for central airway obstruction patients caused by neck and chest tumors to emergency surgery
Source: Sci Rep. 2023 Mar 6;13:3749. doi: 10.1038/s41598-023-30665-1 (PMC9988871; doi:10.1038/s41598-023-30665-1)

# Raw data of case1 CT

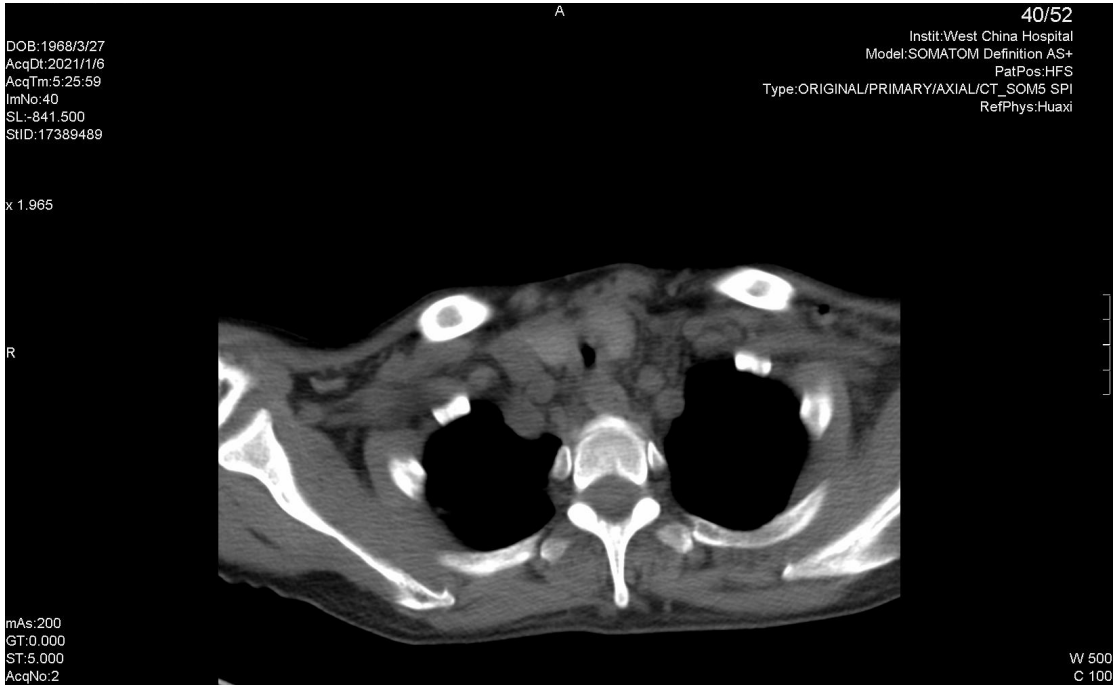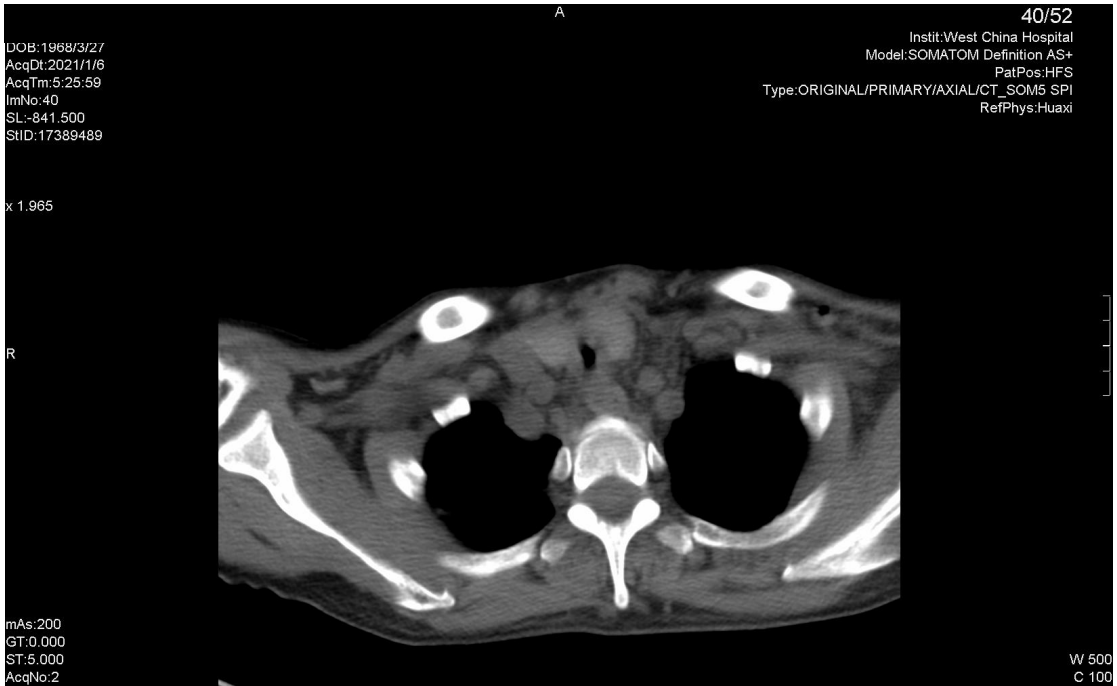

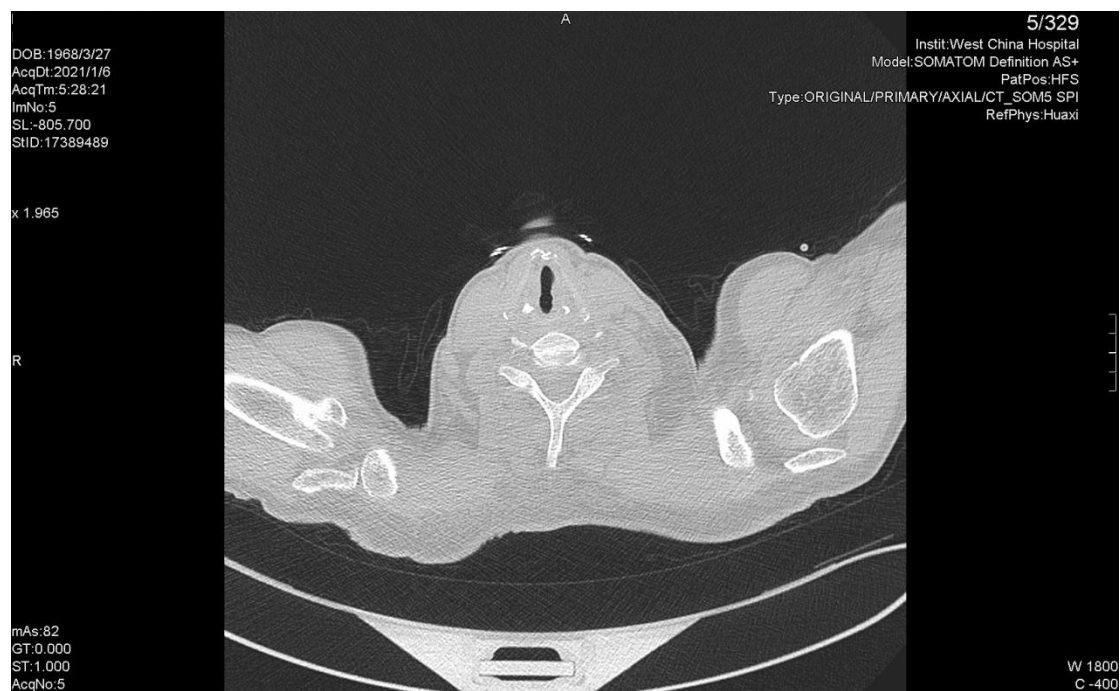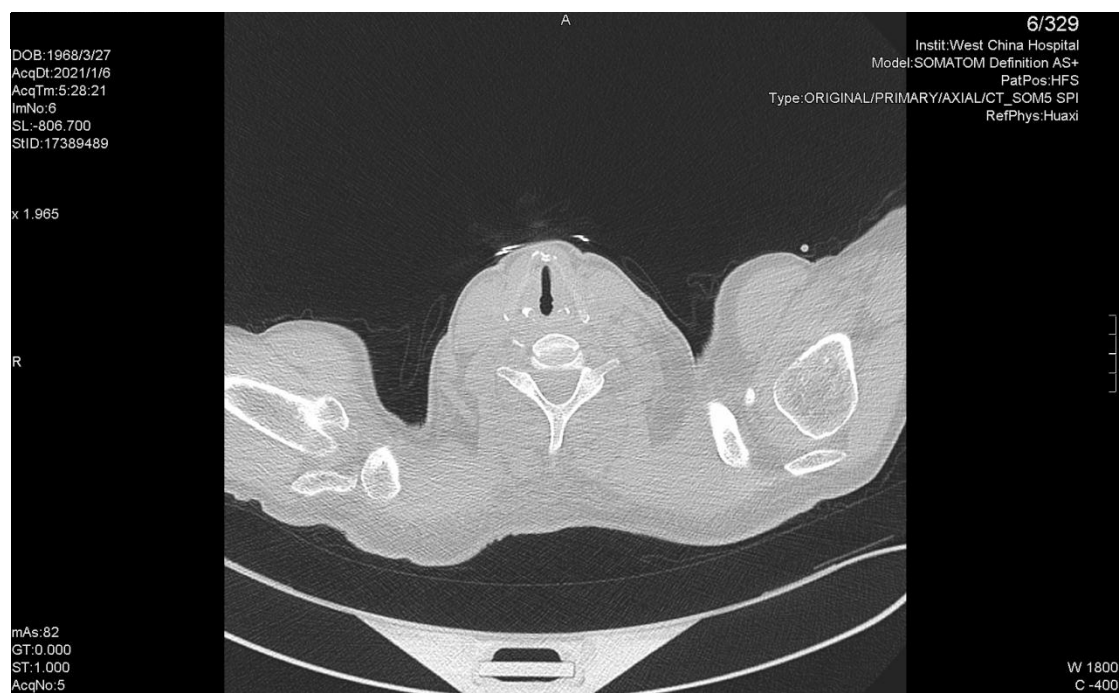

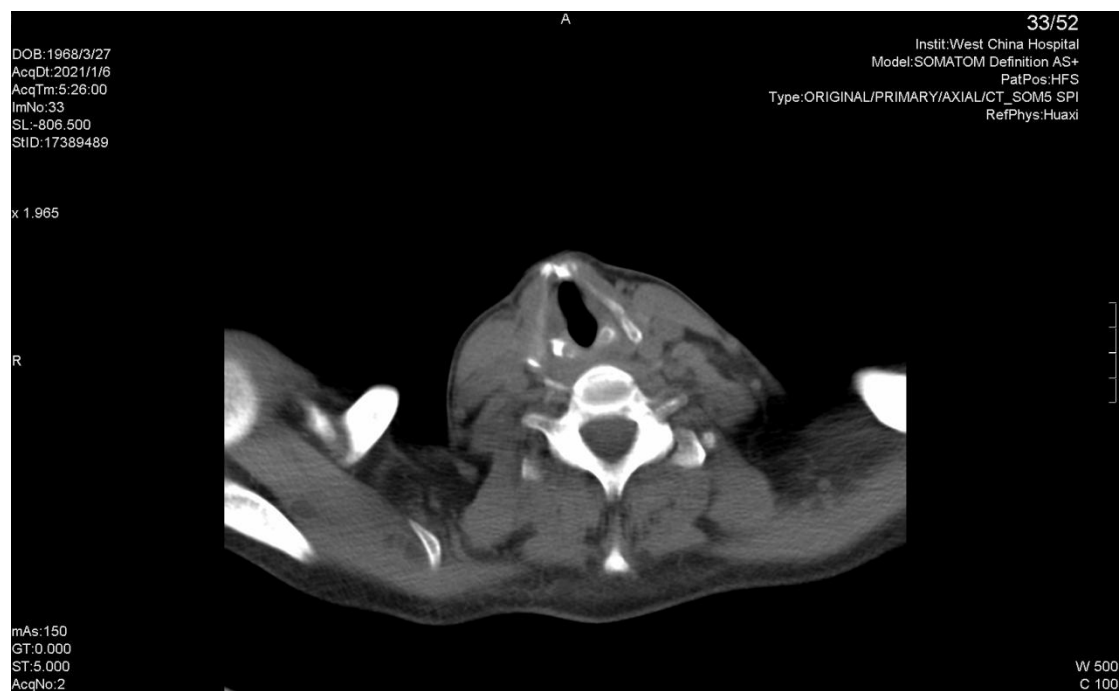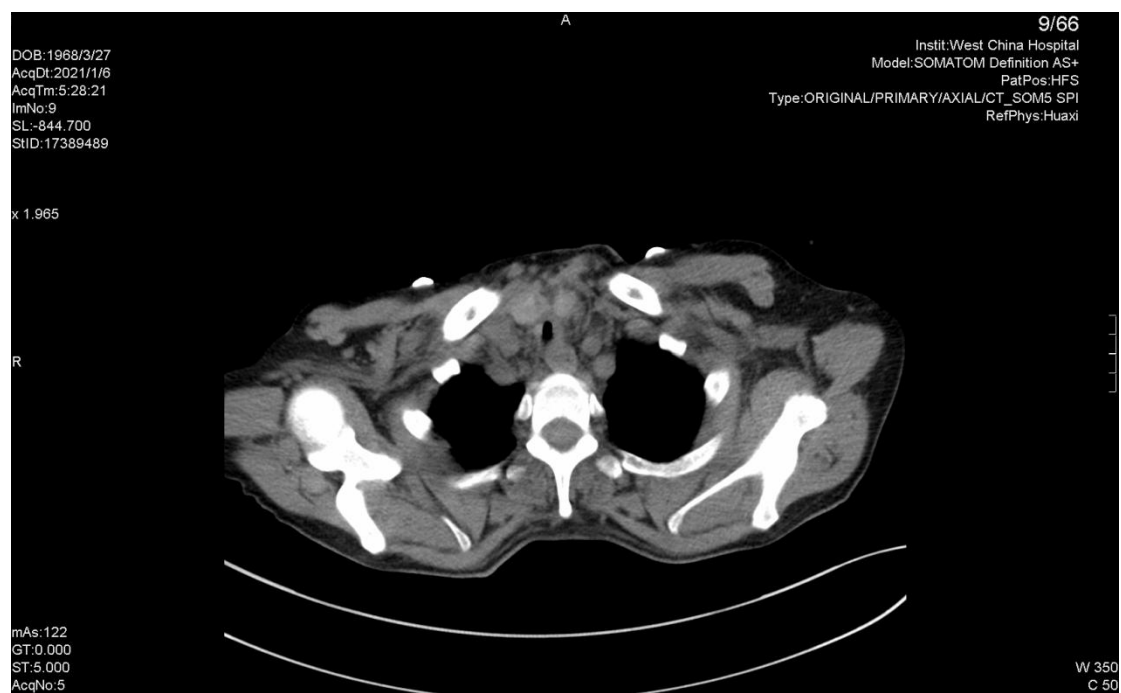

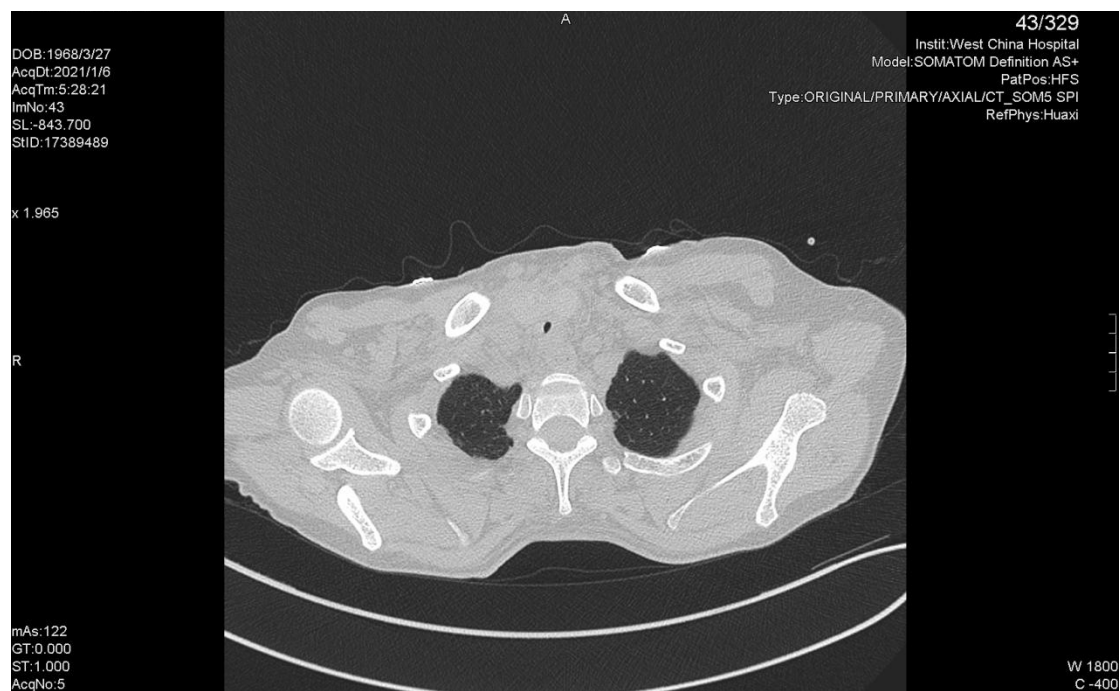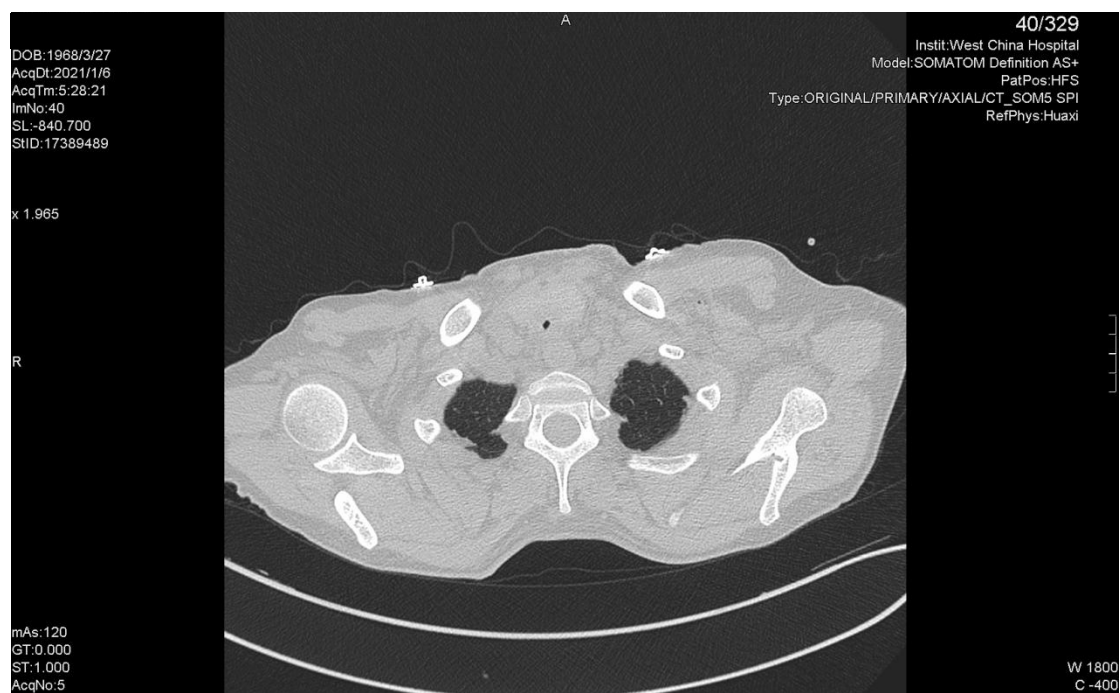

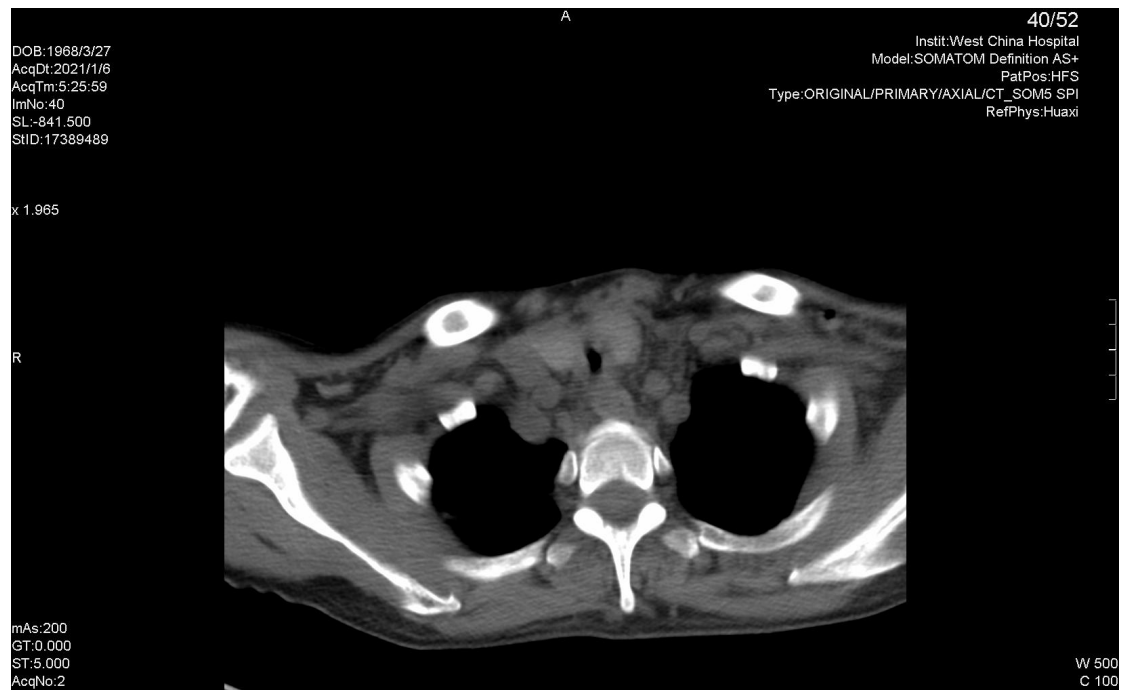

Supplement: Supplementary file 1 — Supplementary Information 1. [file 41598_2023_30665_MOESM1_ESM.pdf]

**Raw data of case1 Pathological examination Hematoxylin-eosin staining**


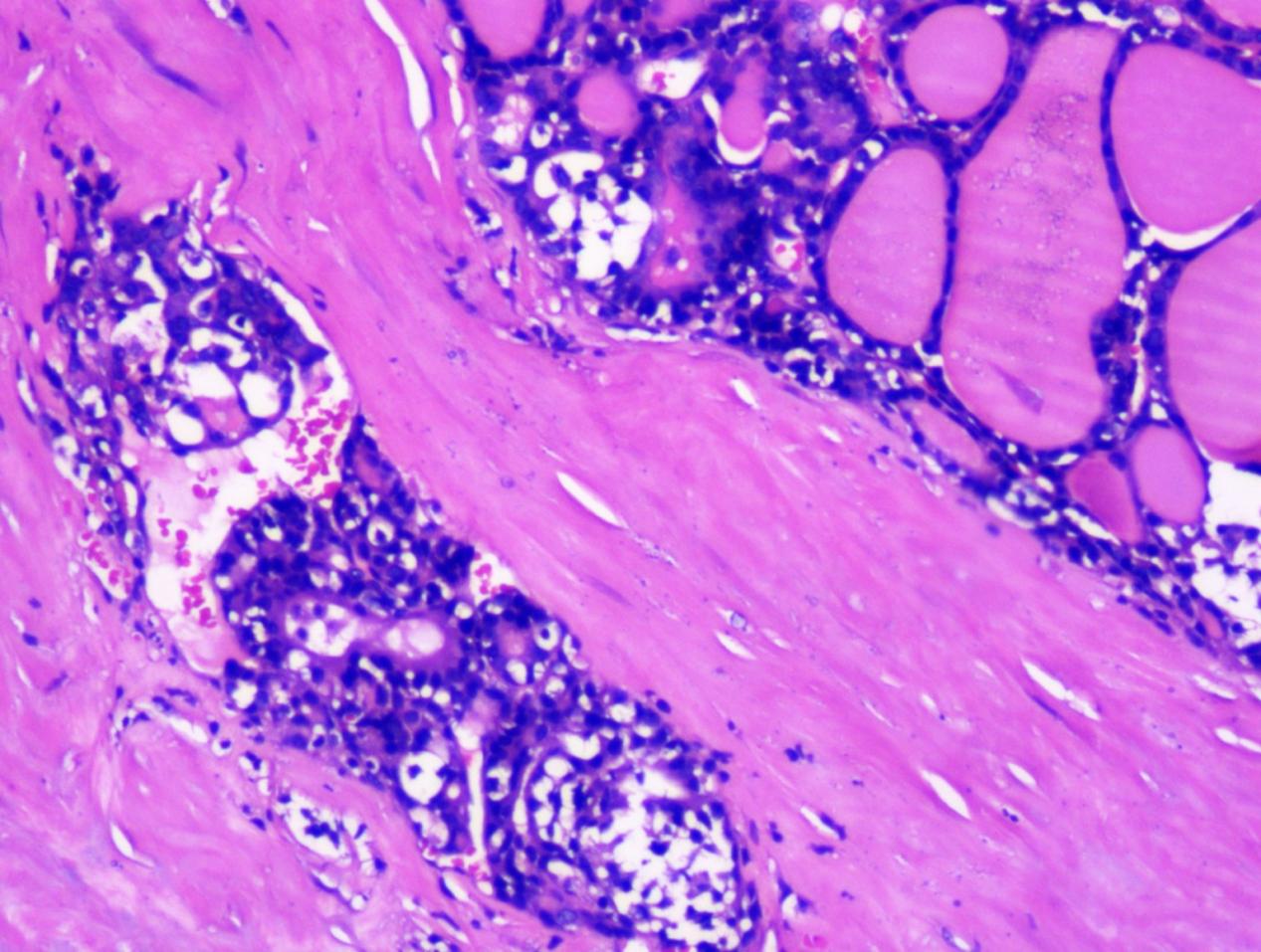


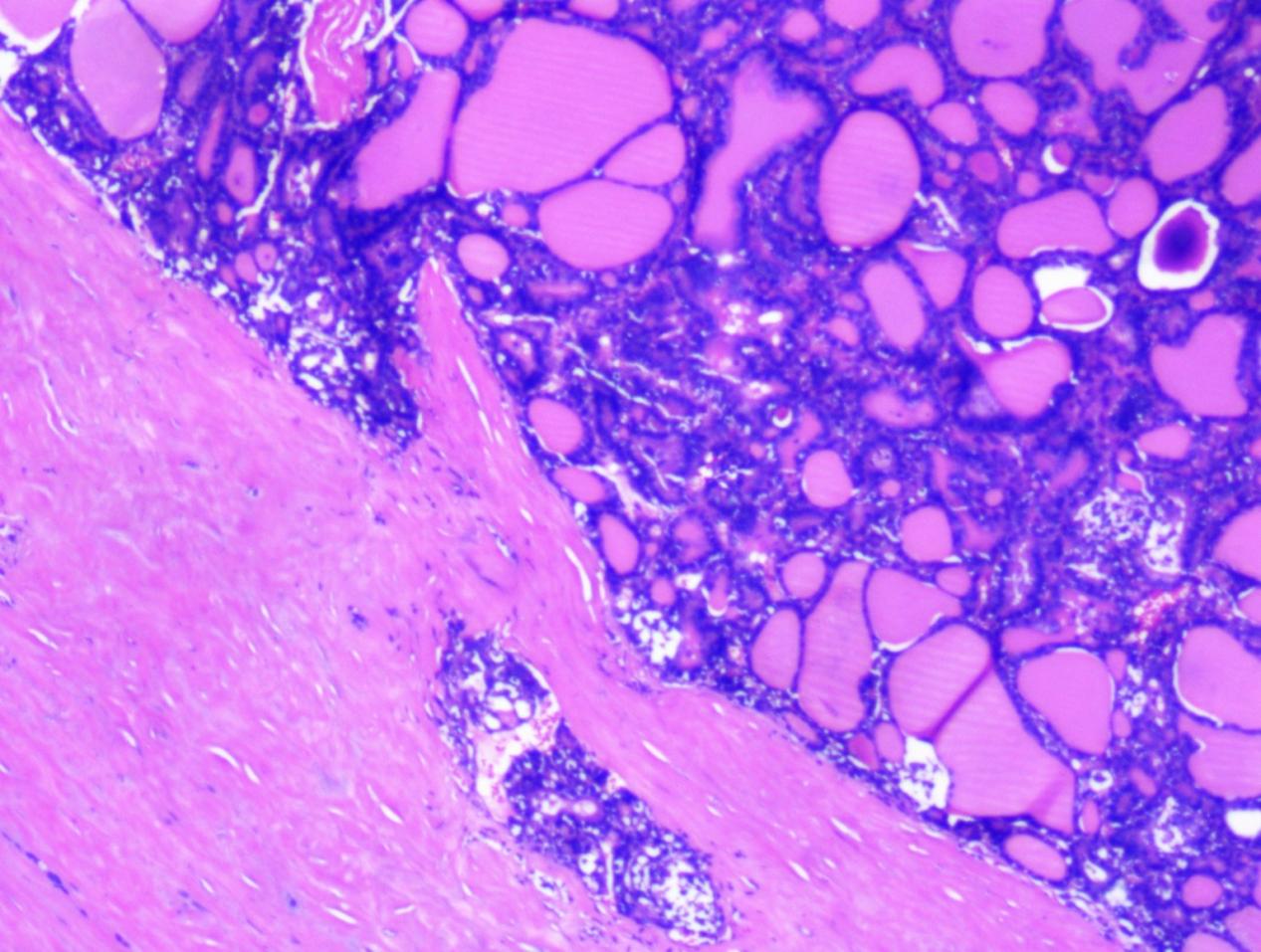

Supplement: Supplementary file 2 — Supplementary Information 2. [file 41598_2023_30665_MOESM2_ESM.docx]

**Raw data of case2 Pathological examination Hematoxylin-eosin staining**


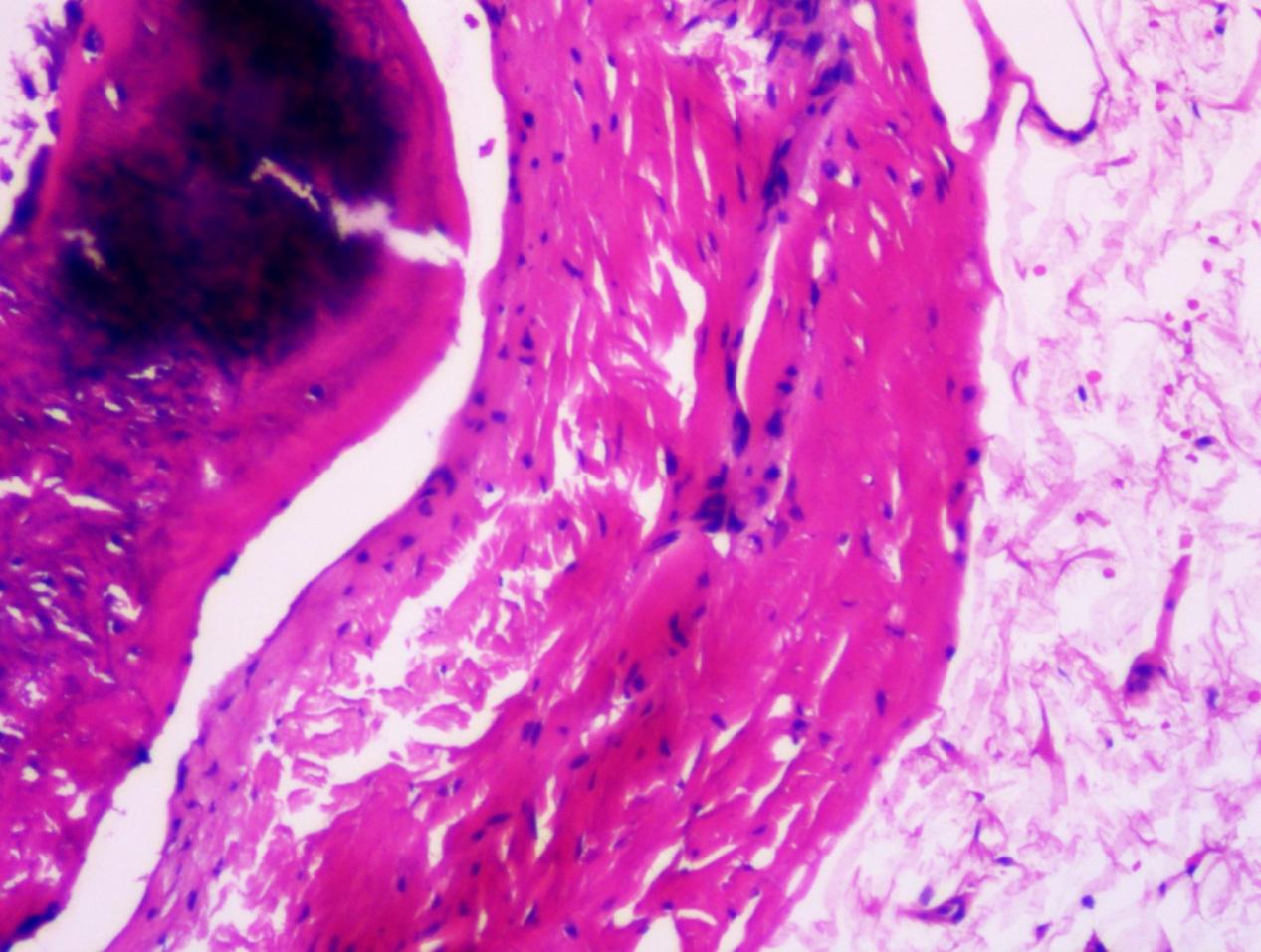


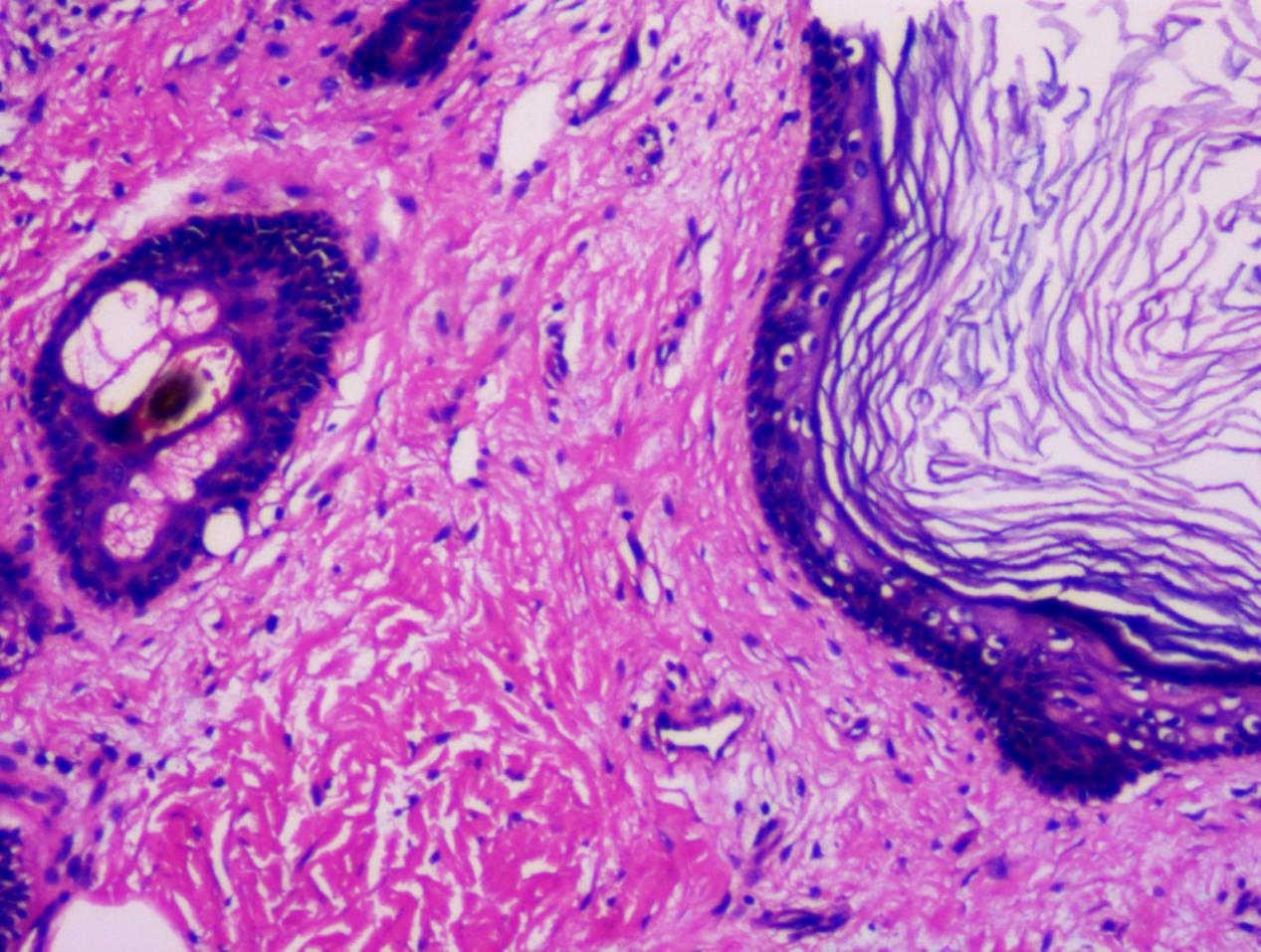

Supplement: Supplementary file 4 — Supplementary Information 4. [file 41598_2023_30665_MOESM4_ESM.docx]

Raw data of case3 CT

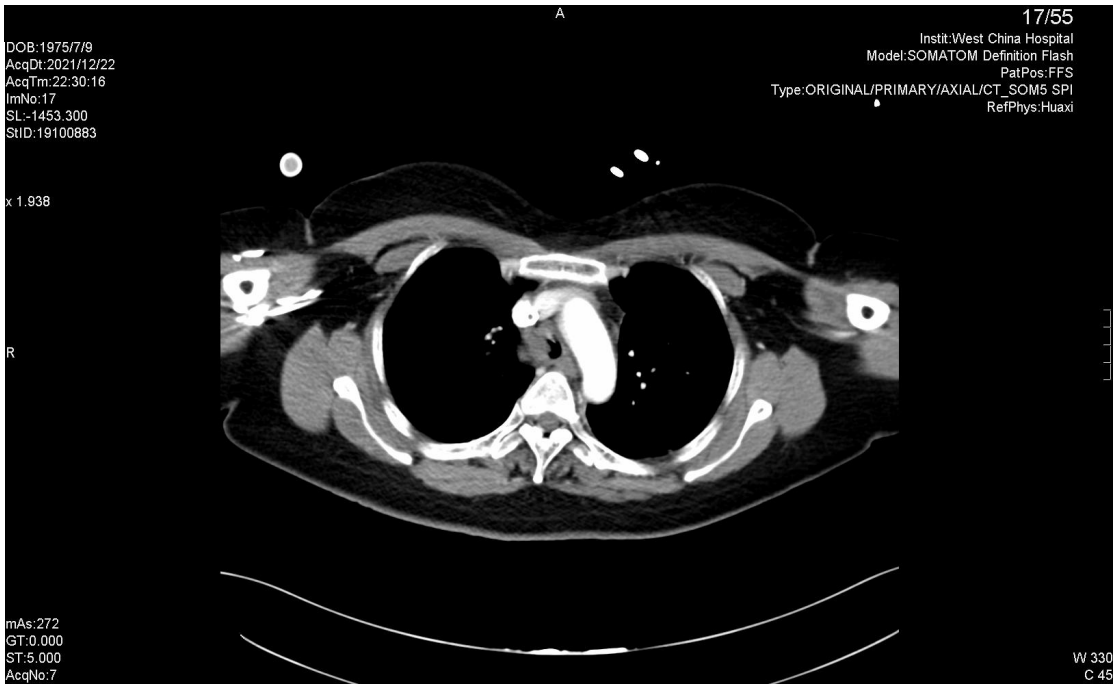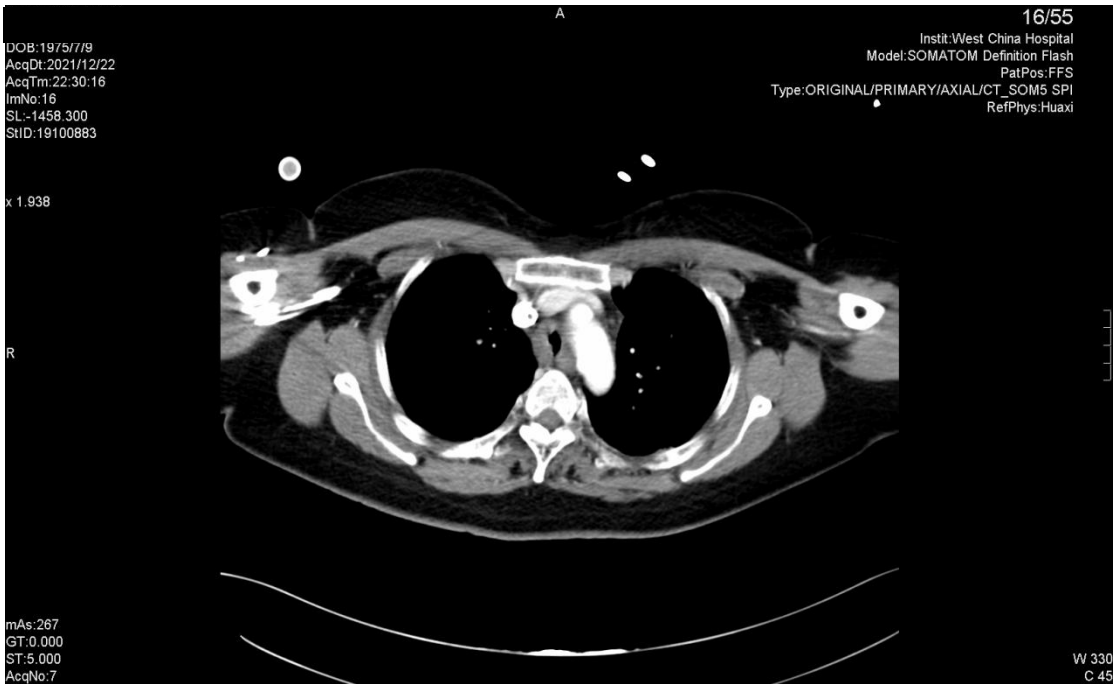

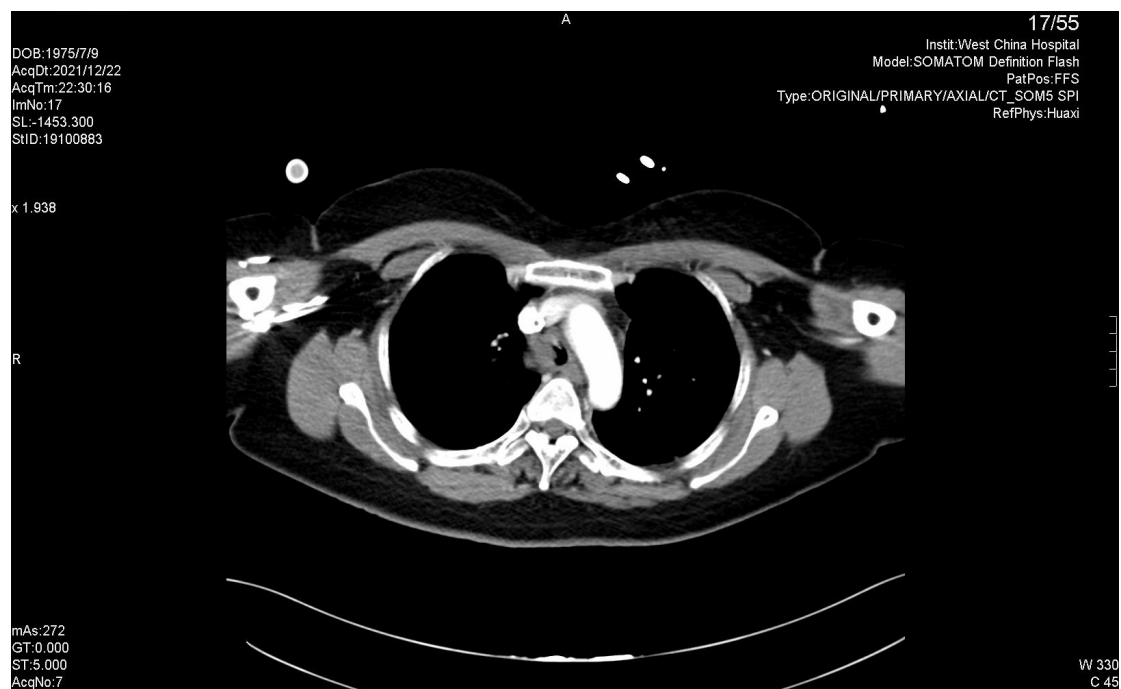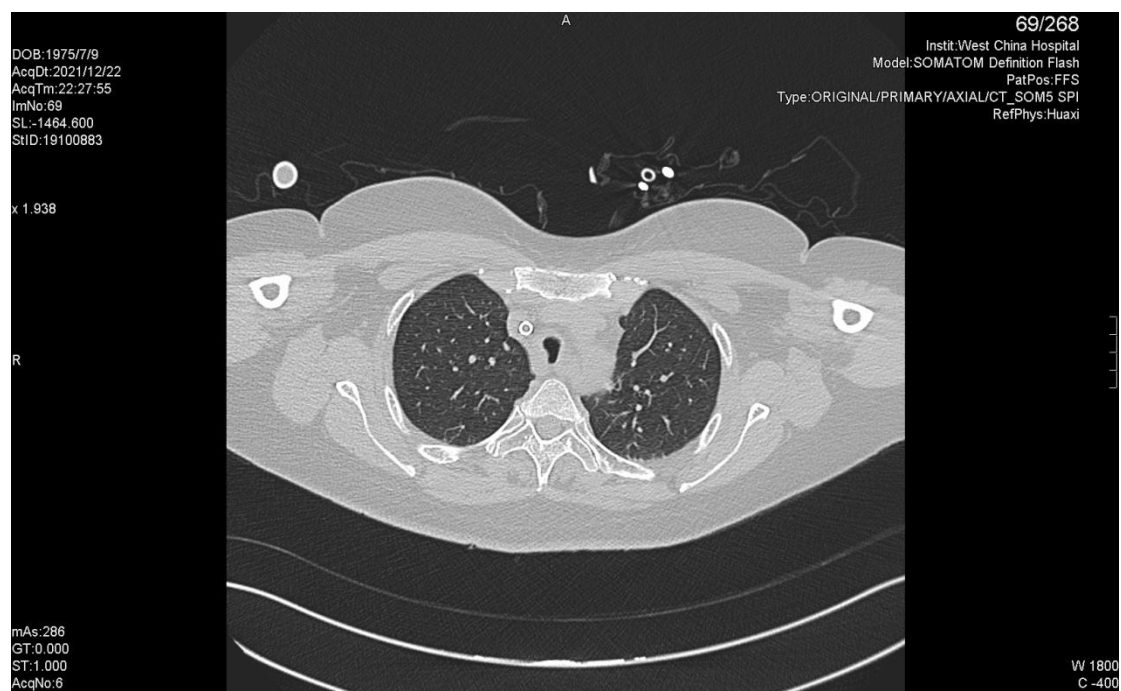

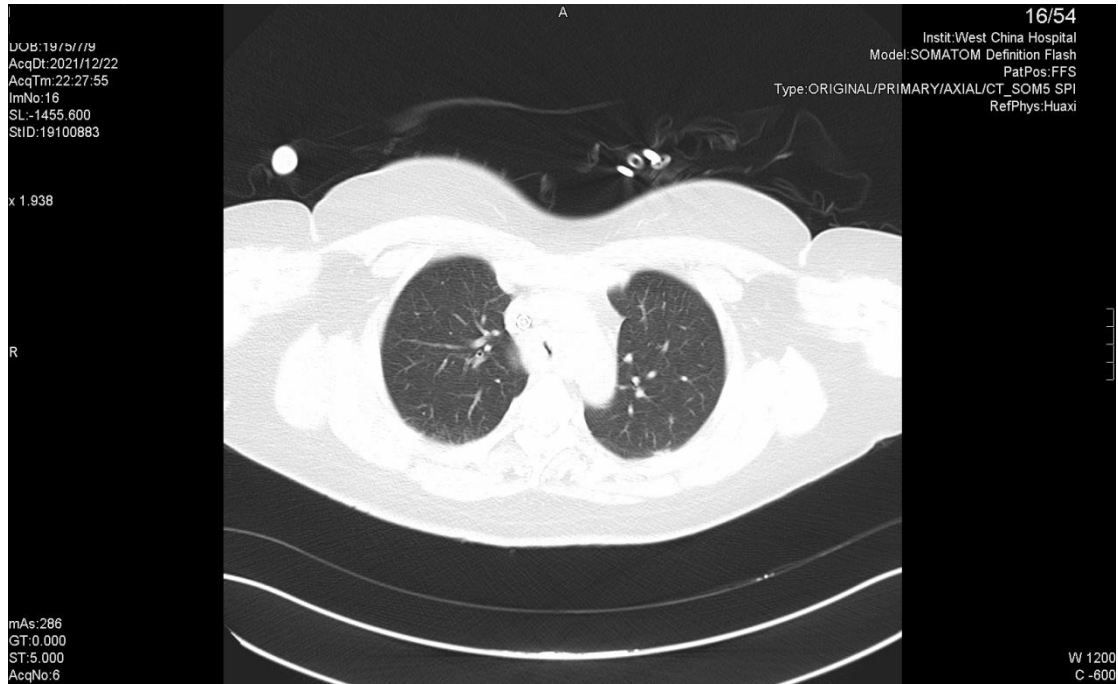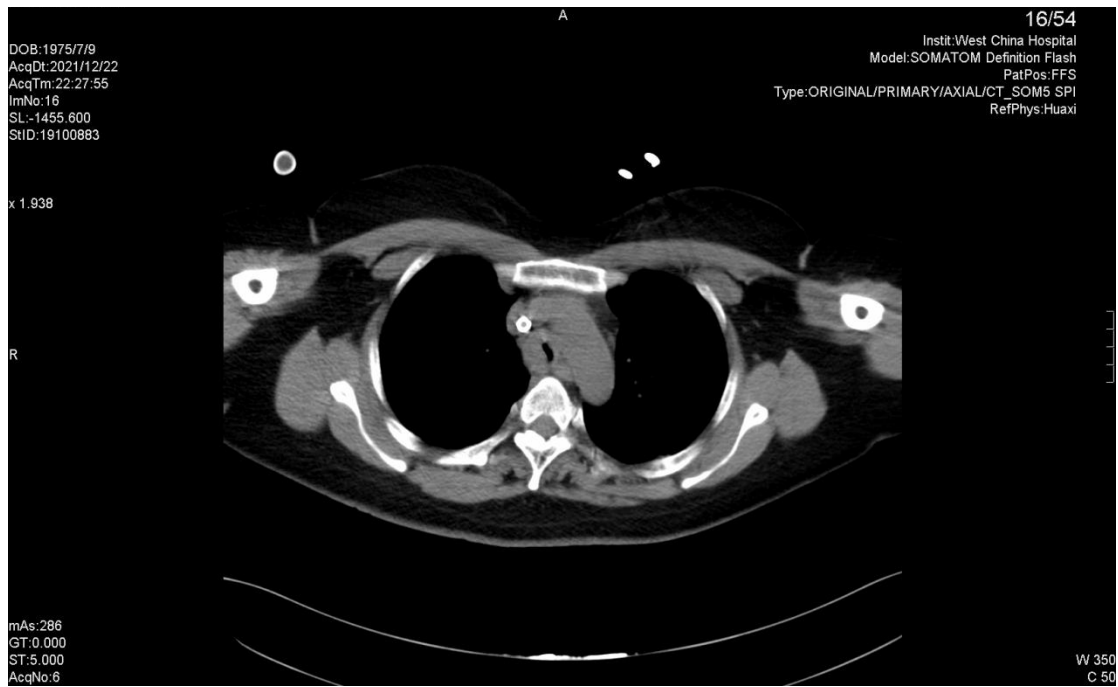

Supplement: Supplementary file 5 — Supplementary Information 5. [file 41598_2023_30665_MOESM5_ESM.pdf]

**Raw data of case3 Pathological examination Hematoxylin-eosin staining**


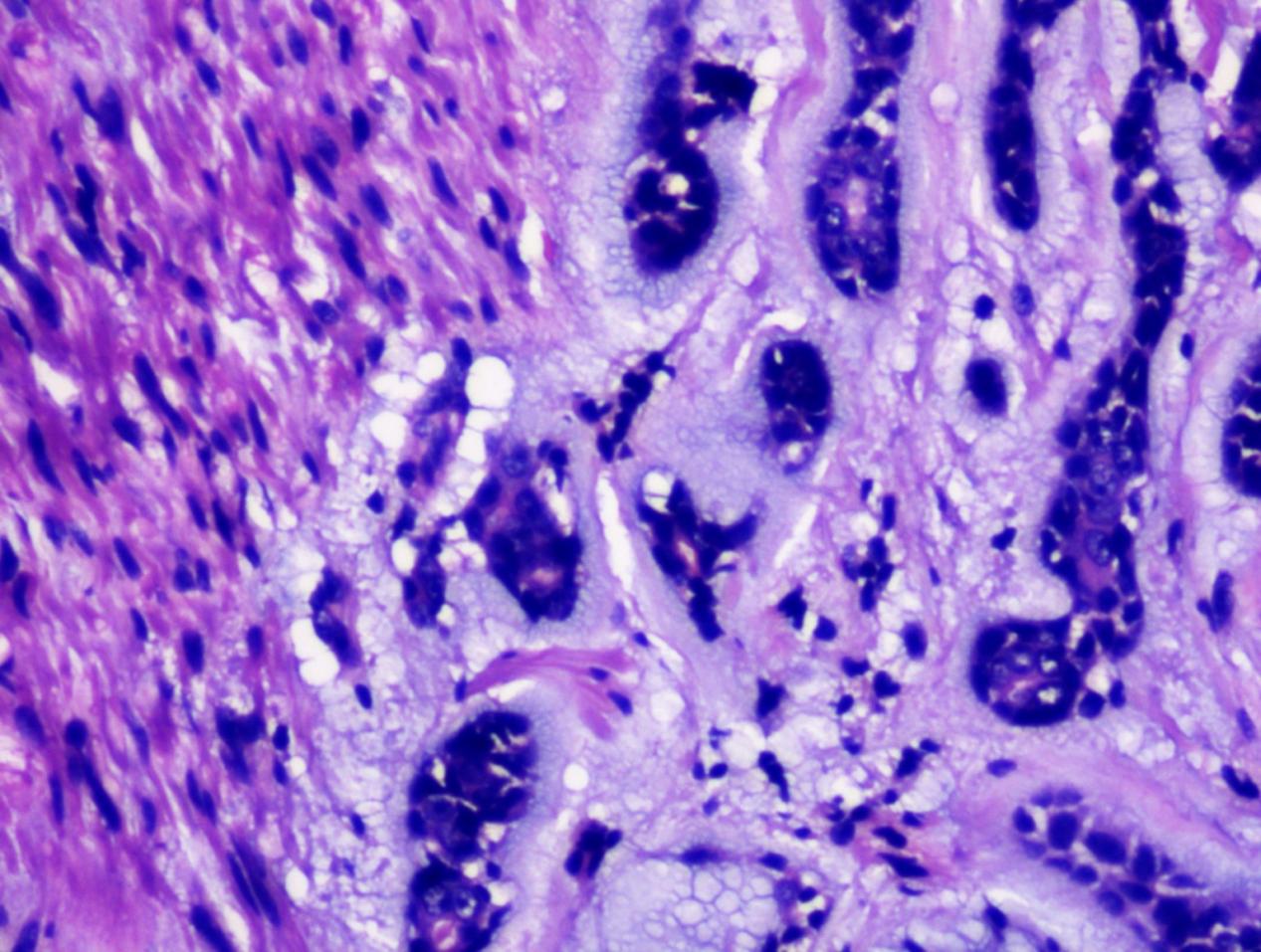


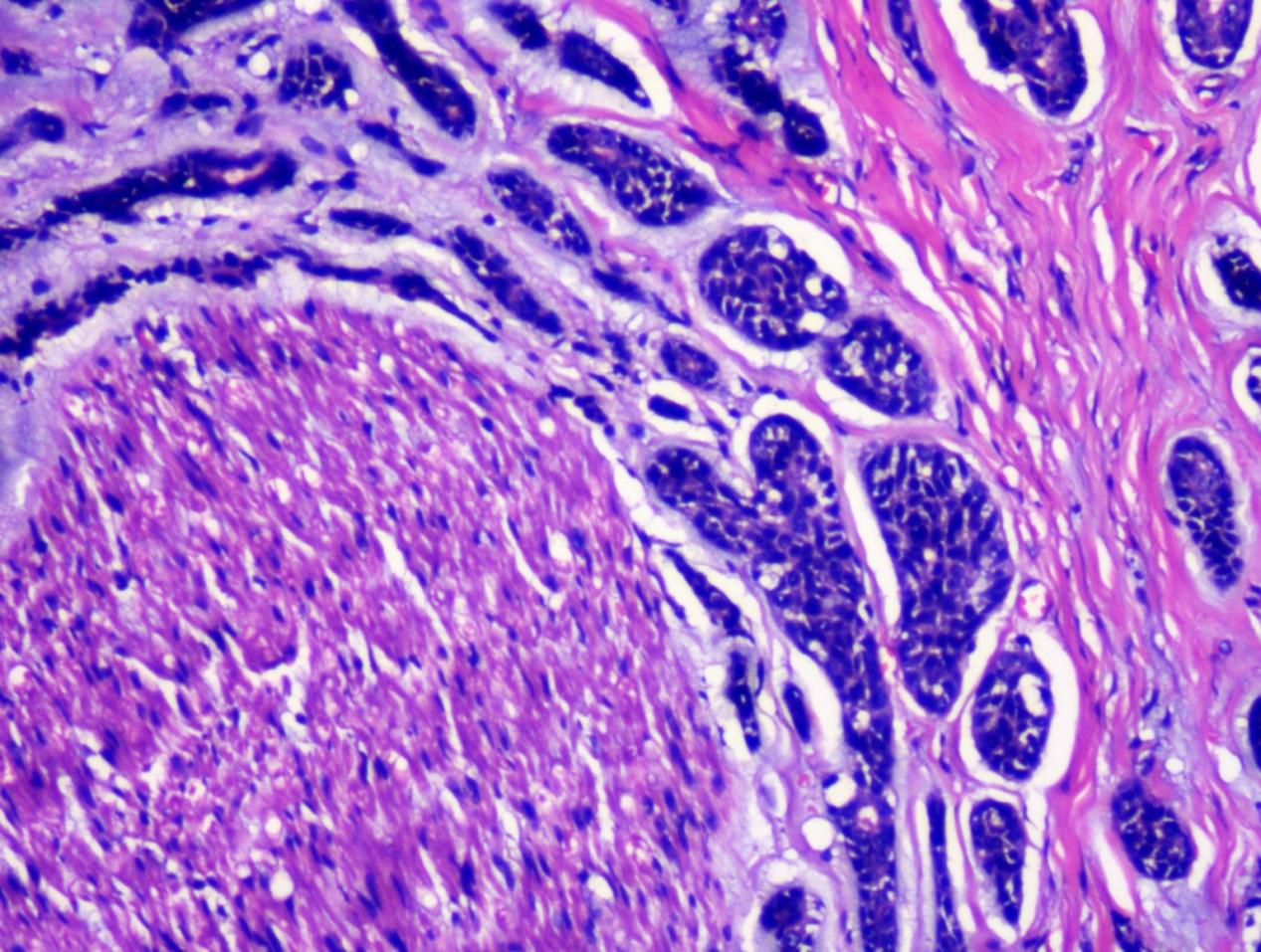

Supplement: Supplementary file 6 — Supplementary Information 6. [file 41598_2023_30665_MOESM6_ESM.docx]
