# Supplementary material for: Early extracorporeal membrane oxygenation as bridge for central airway obstruction patients caused by neck and chest tumors to emergency surgery
Source: Sci Rep. 2023 Mar 6;13:3749. doi: 10.1038/s41598-023-30665-1 (PMC9988871; doi:10.1038/s41598-023-30665-1)
Supplement: Supplementary file 3 — Supplementary Information 3. [file 41598_2023_30665_MOESM3_ESM.pdf]

Raw data of case2 CT

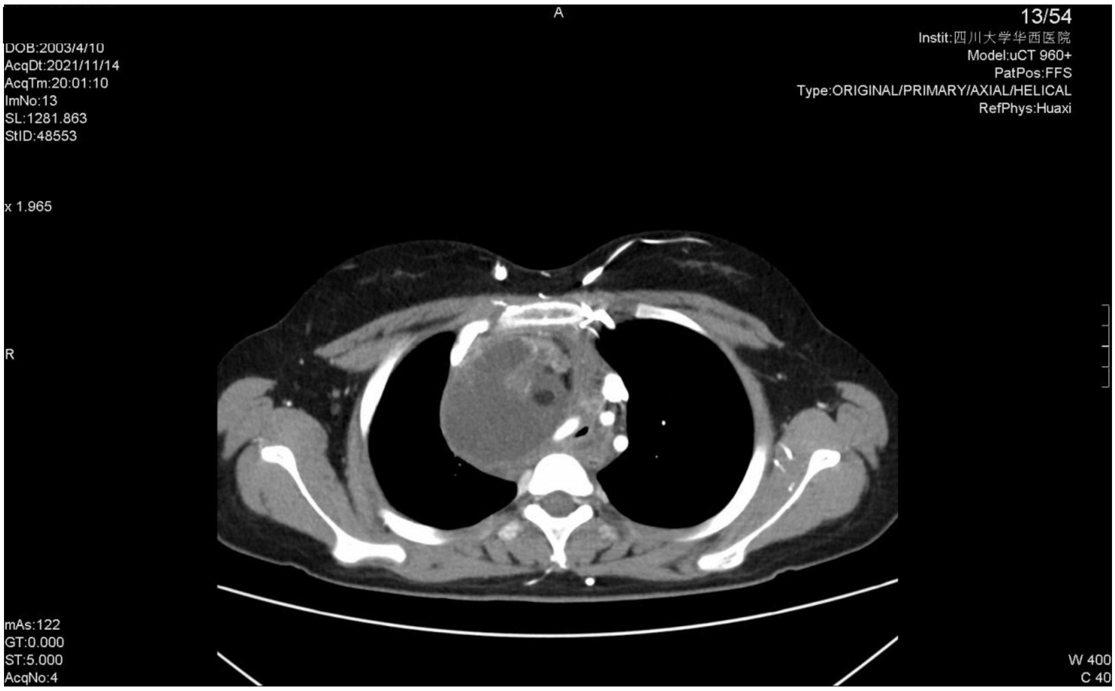

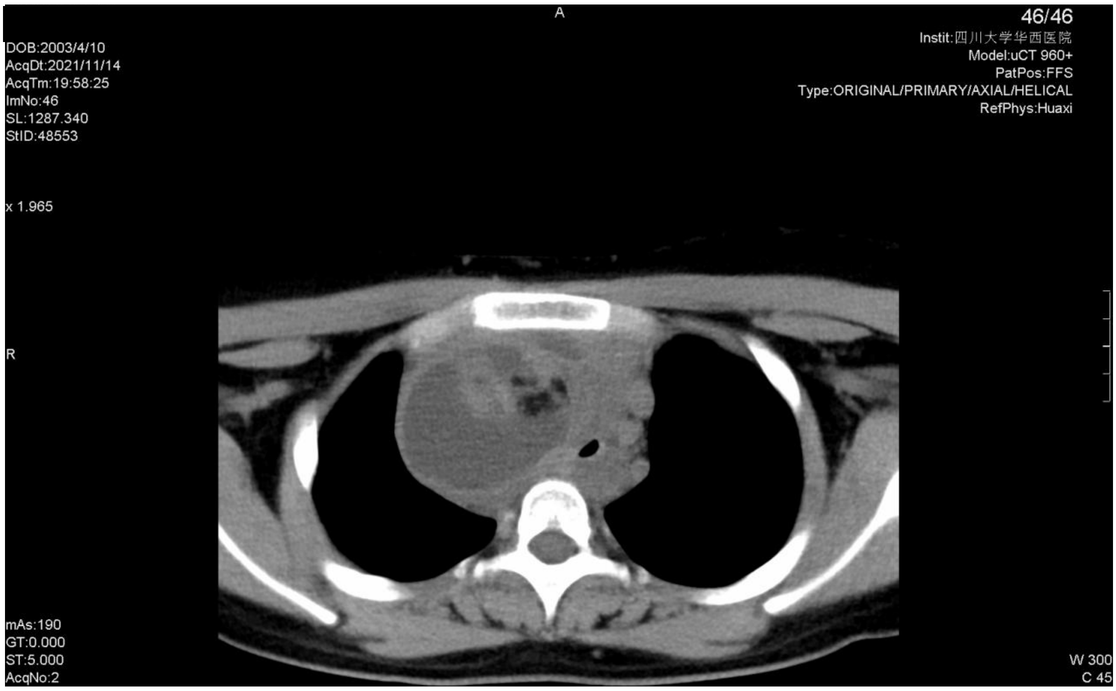

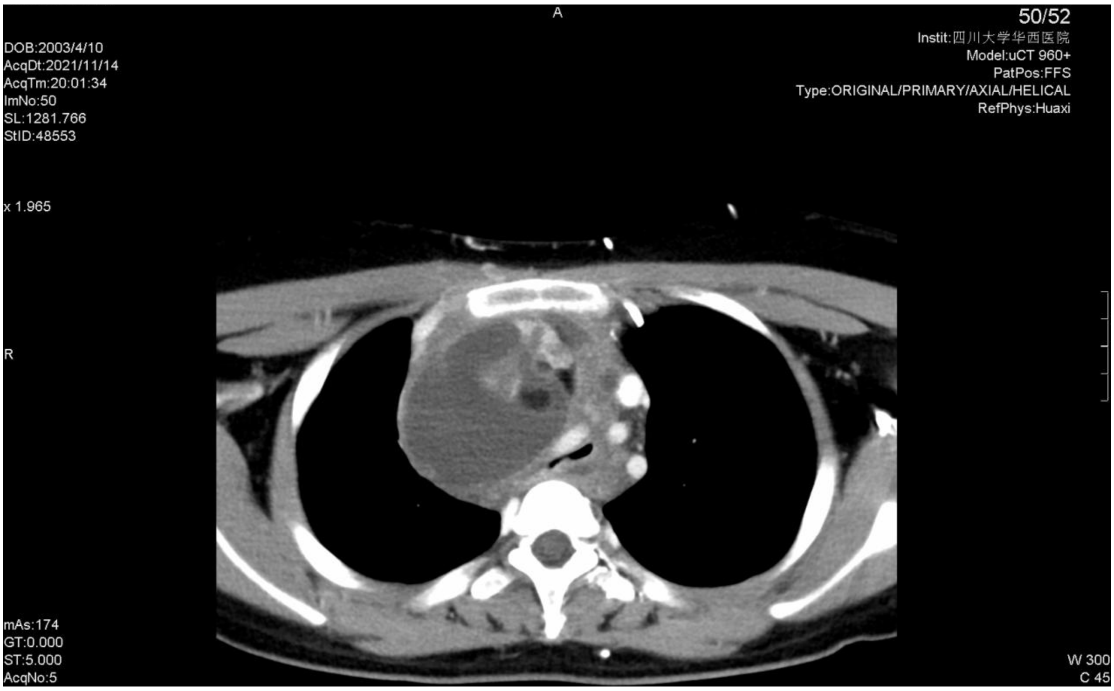
A
50/52
Instit:四川大学华西医院
Model:uCT 960+
PatPos:FFS
Type:ORIGINAL/PRIMARY/AXIAL/HELICAL
RefPhys:Huaxi
DOB:2003/4/10
AcqDt:2021/11/14
AcqTm:20:01:34
ImNo:50
SL:1281.766
StID:48553
x 1.965
R
mAs:174
GT:0.000
ST:5.000
AcqNo:5
W 300
C 45

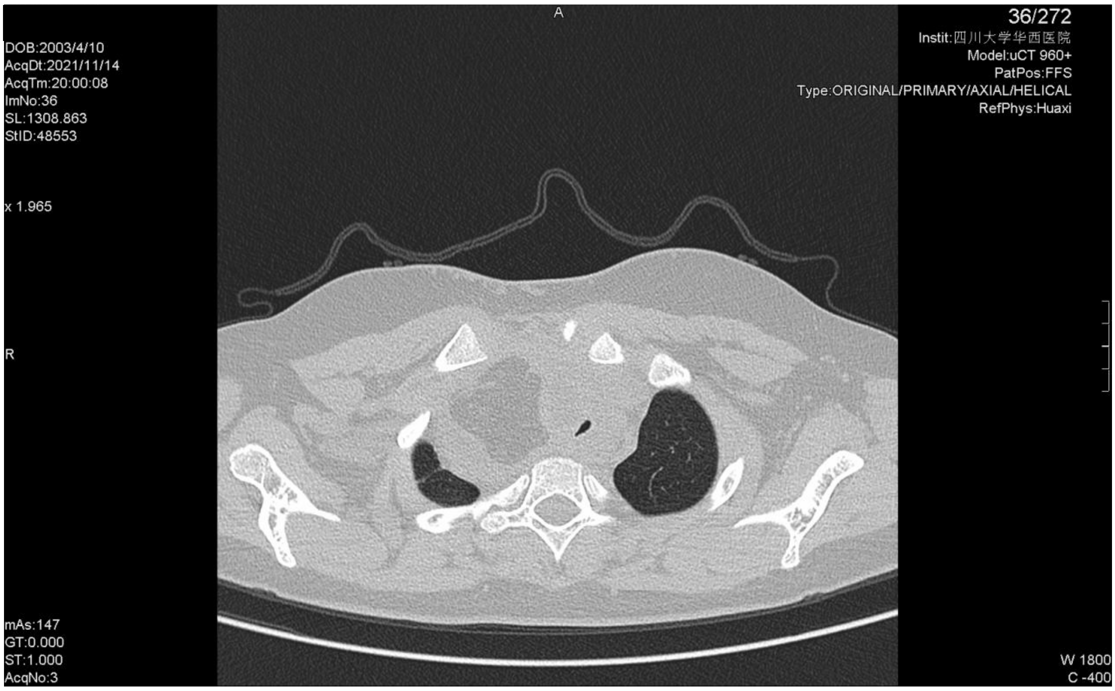
A
36/272
Instit:四川大学华西医院
Model:uCT 960+
PatPos:FFS
Type:ORIGINAL/PRIMARY/AXIAL/HELICAL
RefPhys:Huaxi
DOB:2003/4/10
AcqDt:2021/11/14
AcqTm:20:00:08
ImNo:36
SL:1308.863
StID:48553
x 1.965
R
mAs:147
GT:0.000
ST:1.000
AcqNo:3
W 1800
C -400

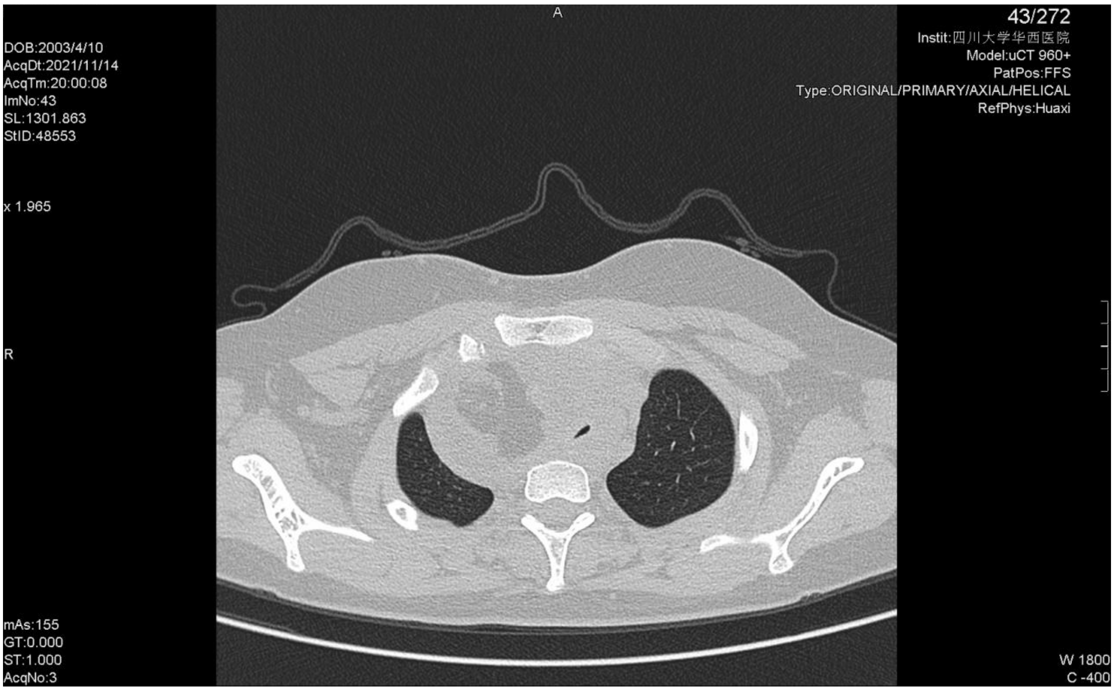
A
43/272
Instit:四川大学华西医院
Model:uCT 960+
PatPos:FFS
Type:ORIGINAL/PRIMARY/AXIAL/HELICAL
RefPhys:Huaxi
DOB:2003/4/10
AcqDt:2021/11/14
AcqTm:20:00:08
ImNo:43
SL:1301.863
StID:48553
x 1.965
R
mAs:155
GT:0.000
ST:1.000
AcqNo:3
W 1800
C -400
